# Effect of freeze-dried *Carica papaya* leaf juice on inflammatory cytokines production during dengue virus infection in AG129 mice

**DOI:** 10.1186/s12906-019-2438-3

**Published:** 2019-02-11

**Authors:** Nor Azrina Norahmad, Mohd Ridzuan Mohd Abd Razak, Norazlan Mohmad Misnan, Nur Hana Md Jelas, Umi Rubiah Sastu, Amirrudin Muhammad, Tiffiny Chau Dee Ho, Bazilah Jusoh, Nor Azlina Zolkifli, Ravindran Thayan, Adiratna Mat Ripen, Murizal Zainol, Ami Fazlin Syed Mohamed

**Affiliations:** 1 Herbal Medicine Research Center, Institute for Medical Research, Ministry of Health Malaysia, 50588 Kuala Lumpur, Malaysia; 2 Infectious Disease Research Center, Institute for Medical Research, Ministry of Health Malaysia, 50588 Kuala Lumpur, Malaysia; 3 Allergy and Immunology Research Center, Institute for Medical Research, Ministry of Health Malaysia, 50588 Kuala Lumpur, Malaysia

**Keywords:** *Carica papaya*, AG129, Dengue, Cytokine, Gene expression, In vivo

## Abstract

**Background:**

*Carica papaya* leaves have been used for traditional treatment of dengue fever and have been reported to exhibit an immunomodulatory activity by affecting the level of cytokine production in vitro and in vivo. Due to the lack of adequate in vivo evidence in dengue disease model, the present study was initiated to screen and identify the cytokines affected by freeze-dried *C. papaya* leaf juice (FCPLJ) treatment in AG129 mice infected with DEN-2 dengue virus.

**Methods:**

The AG129 mice were fed orally with FCPLJ for 3 consecutive days after 24 h of dengue virus inoculation. Plasma cytokines were screened by using ProcartaPlex immunoassay. The gene expression in the liver was analyzed by using RT^2^ Profiler PCR Array.

**Results:**

The results showed that FCPLJ treatment has increased the plasma CCL2/MCP-1 level during peak of viremia. Gene expression study has identified 8 inflammatory cytokine genes which were downregulated in the liver of infected AG129 mice treated with FCPLJ. The downregulated inflammatory cytokine genes were CCL6/MRP-1, CCL8/MCP-2, CCL12/MCP-5, CCL17/TARC, IL1R1, IL1RN/IL1Ra, NAMPT/PBEF1 and PF4/CXCL4.

**Conclusion:**

The findings indicated the possible immunomodulatory role of FCPLJ during dengue virus infection in AG129 mice.

**Electronic supplementary material:**

The online version of this article (10.1186/s12906-019-2438-3) contains supplementary material, which is available to authorized users.

## Background

Dengue is rapid emerging mosquito-borne viral disease, affecting countries in tropical and subtropical regions around the world. Globally, it is estimated that there are 390 million dengue infection occur each year [1]. The virus is being transmitted through the bites of female *Aedes* mosquito infected with one of four dengue virus serotypes (DEN-1, DEN-2, DEN-3 and DEN-4). For a healthy individual, the first symptom of the disease appears 3–7 days after being bitten by an infected mosquito. The symptoms ranged from mild to high fever, severe headache with muscle and joint pain. The dengue hemorrhagic fever is characterized by fever, hemorrhagic sign, thrombocytopenia and plasma leakage [2]. The pathogenesis of dengue infection involves host-specific immune responses, including immune cell activation, secretion of cytokines and chemokines, the production of inflammatory mediators, and autoimmunity [3]. For example, high level of proinflammatory cytokines such as TNF-α, IL-6 and IL-8 were observed during delayed viral clearance that results in endothelial activation and vascular leakage [4].

In the absence of antiviral drug to treat the disease, various alternative treatments are being explored including *C. papaya* leaf juice (CPLJ)*.* Many scientists have investigated the possible use of this plant as supportive treatment for dengue patients. The beneficial effects of CPLJ and extracts in increasing the platelet level in rodents have been studied extensively [5–8]. For the past 10 years, a number of clinical trial and clinical case studies on the platelet increasing property of CPLJ or extracts in dengue patient have been reported [9–13].

The *C. papaya* leaf extract and juice were also studied for their antioxidant and immunomodulatory activities in vitro [14, 15] and in vivo [6, 16, 17]. The leaves extract has been shown to regulate certain cytokine production in activated human peripheral blood mononuclear cells (PBMCs) in vitro [14, 15, 18]. An in vivo study has shown that *C. papaya* leaf extract treatment reduced the inflammatory effect in paw oedema induced rats [6]. In addition, the CPLJ treatment has also reduced the TNF-α production, increased the phagocytic index and prevented the reduction of leucocyte count in cyclophosphamide-induced neutropenia rats [16]. Moreover, white blood cell count, bone marrow cell count, splenocyte count and peritoneal macrophages phagocytic activity were increased in healthy rats treated with CPLJ [17].

In the present study, we looked at the potential of freeze-dried *C. papaya* leaf juice (FCPLJ) as part of dengue treatment by investigating its effects on inflammatory cytokines in the plasma of dengue virus infected AG129 mice. In addition, we also analyzed the gene expression profiles of 84 mouse inflammatory cytokines & receptors that would be affected by the FCPLJ treatment in the liver of dengue virus infected AG129 mice.

## Methods

### FCPLJ preparation

Fresh and healthy green leaves of *C. papaya* were collected from the herbal garden of the Institute for Medical Research, Kuala Lumpur, Malaysia. The plant was identified by Ms. Tan Ai Lee, a botanist from Forest Research Institute Malaysia, Kepong, Malaysia (FRIM). A voucher specimen was deposited at the FRIM (Voucher No: 007/10). The leaves were cleaned thoroughly with veggie wash to remove any contaminant and rinsed with reverse osmosis water. The juice from the leaves was extracted by using a juicer and the remaining husk was pressed using a clean laboratory cloth. The juice was placed in containers and kept frozen at − 50 °C prior to freeze drying process (Virtis Advantage, SP Scientific, USA). The green powder was kept at 4 °C until further use. The powder was dissolved in distilled water prior to dosing.

### Analytical high performance liquid chromatography (HPLC) analysis

The flavonoid compounds; nicotiflorin and rutin were purchased from Extrasynthese (Genay, France) whereas clitorin and manghaslin were isolated from *C. papaya* leaf extract and the structural elucidation were compared to previous publish data [19–21]. Analysis of HPLC was performed to monitor these flavonoid compounds in *C. papaya* leaf samples on a Waters 2695 HPLC system (Milford, USA) equipped with a Waters 996 photodiode array detector and a Waters 2695 separation module with empower 2.0 software. A Waters Symmetry C18 Column, 300 Å, 5 μm, 3.9 mm X 150 mm, (Milford, USA) was used as stationary phase and the mobile phase consisted of water containing 0.01% Trifluoroacetic acid (TFA) (Solvent A) and acetonitrile containing 0.01% TFA (Solvent B). The gradient solvent system of solvent A and solvent B as follows: 90% solvent A until 5 min, followed by 90–750% solvent A over 20 min, then 75–5% A over 5 min, then going back to 90% solvent A until 5 min, and finally reconditioning the column with 90% solvent A isocratic for 5 min. The flow rate for this analysis was maintain in 1.0 ml/min and the injection volume were 20 μl. The chromatogram was monitored using a wavelength range of 210–400 nm.

### Propagation of viruses for inoculation

The dengue virus propagation was prepared according to procedures described by McCormich et al., 2012 [22] with some modification. The New Guinea C, a DEN-2 strain, was propagated in C6/36 mosquito cells in Leibovitz’s L-15 medium supplemented with 2% fetal bovine serum, 10% tryptose phosphate broth, 100 U/ml penicillin G and 100 μg/ml streptomycin at 28 °C. For quantification of virus, 4.5 X 10^5^ cells/well of Vero cells were grown in each well of a 6-well plate containing 1.5 ml Dulbecco’s modified Eagle’s medium (DMEM) for 24 h until confluent. Then, the media overlaying the monolayer was discarded and 100 μl of cell supernatant containing virus suspension were allowed to adsorb for one hour. One ml of an overlay medium containing 1% agarose in 1X DMEM medium was added to cell monolayer and incubated for 7 days at 37 °C in 5% CO_2_ incubator. The uninfected cells were stained blue with 1% crystal violet in 20% ethanol leaving clear plaques of infected cells on the monolayer. The infectivity titre is expressed as the number of plaque forming units per ml (PFU/ml).

### Experimental animal and dengue viruses inoculation

Four weeks old, male AG129 mice (129/Sv mice deficient in both alpha/beta and gamma interferon receptors) were procured from Marshall BioResources, United Kingdom. Upon arrival, all mice were quarantined for 2 weeks and acclimatized for 1 week under a well-ventilated environment in Non-clinical Research Facility, Institute for Medical Research, Kuala Lumpur, Malaysia. The mice were housed in individual ventilated cages supplied with reverse osmosis drinking water and mouse pellet ad libitum.

The mice were divided into two experiments; plasma cytokine screening and gene expression analysis. For each experiment, the mice were divided into 3 groups of five: mock infected (uninfected; N = 5); infected (N = 5) and infected with FCPLJ treatment (N = 5). The mice were inoculated with 2X10^6^ PFU DEN-2, New Guinea C strain, in 0.2 ml volume intraperitoneally for infected group and 0.2 ml plain media for mock infected group.

After dengue virus inoculation through intraperitoneal route, the body weight of the mice were recorded daily. The sign of illness was visually monitored twice a day during the experiment. The sign of illness was scored based on 1 to 5 scale: 1-healthy; 2-mild sign of lethargy and ruffled fur; 3-intermediate level of lethargy, ruffled fur and hunched posture; 4-very lethargy, ruffled fur, hunched posture and limited mobility; 5-moribund with limited to no mobility and inability to reach food or water [23]. Mice exhibiting weight loss more than 20% of initial body weight or moribund or paralyzed during the study were euthanized immediately [24] by open-drop exposure to 5% isoflurane administered in 1 L chamber volume. None of the mice died before meeting criteria for euthanasia. At the end of the study the survived mice were euthanized by open-drop exposure to isoflurane. The euthanization process was done in fume hood by the veterinarian.

### Dose determinations

The dose was selected based on general toxicology studies conducted by Institute for Medical Research, Malaysia [19, 25]. No mortality and adverse effect on the functions of the liver, kidney and bone marrow was observed in rats treated with up to 2000 mg/kg BW FCPLJ. For cytokine screening, the treatment group was administered with daily doses of 500 mg/kg BW FCPLJ by oral route for 3 consecutive days. The mock infected and infected only groups were given distilled water. A second experiment was conducted for a concentration of 1000 mg/kg BW FCPLJ. For gene expression study, a similar group of mice were set up and the treatment group was given 1000 mg/kg BW of FCPLJ. General behavior and clinical signs were recorded daily. All experiments involving mice were performed in compliance with the guidelines of the Animal Care and Use Committee, Ministry of Health Malaysia (ACUC-MOH), ACUC/KKM/02(9/2016).

### Sample collections

Two hundred microlitres of whole blood was collected in EDTA microtainer tube on day 3, 5 and 7 post-infection by submandibular vein puncture technique. Blood smear was prepared from 2 μl of whole blood and stained with Giemsa for blood differential count. For cytokine screening, plasma was separated from the whole blood by centrifugation for 15 min at 2000 x g at 4 °C. The plasma was immediately transferred into a clean 1.5 ml microcentrifuge tube and stored at − 80 °C prior to be used for cytokine screening. For gene expression analysis, mice were euthanized on day 4 post-infection. Whole blood was collected by cardiac puncture directly in a syringe and transferred into EDTA microtainer tube. The blood was centrifuged for 15 min at 2000 x g at 4 °C to obtain the plasma for the purpose of NS1 assay. Livers were harvested and kept in RNAlater™ Stabilization Solution (Invitrogen, USA). The livers were kept at − 20 °C until further use.

### Detection of NS1 production during dengue infection in AG129 mice

The NS1 antigen detection in infected AG129 mice was done by using Platelia Dengue NS1 Ag kit (Bio-Rad Laboratories, USA). Plasma was diluted 1:10 with 1X PBS prior to the assay. Briefly, 50 μL of sample and controls were diluted 1:2 with sample diluent and combined with 100 μL of conjugate (anti-NS1 murine monoclonal antibodies (MAb) with horseradish peroxydase antibody). This solution was added to microtiter plates coated with anti-NS1 monoclonal antibodies and incubated at 37 °C for 90 min followed by washing. When NS1 antigen was present, an immune-complex MAb-NS1-MAb-peroxidase formed and was exposed by adding a chromogenic substrate (tetramethylbenzidine and H_2_O_2_) to initiate color development. The reaction was halted by the addition of 100 μL of stopping solution containing 1 N sulfuric acid and the optical density of samples was read at 450 nm using FLUOstar® Omega microplate reader (BMG Labtech, Germany).

### Cytokine screening and analysis

A customized ProcartaPlex™ Immunoassay Mouse Cytokine 12-plex (CSF3/G-CSF, CSF2/GM-CSF, IFN-γ, IL-1β, IL-4, IL-6, IL-10, IL-13, CCL2/MCP-1, TNF-α, CCL3/MIP-1A and IL-18) was used to screen cytokine in 50 μl of plasma according to manufacturer’s instructions. Values for CSF2/GM-CSF, IL-1β, IL-4, IL-10 and IL-13 were outside the detectable range for a majority of the experimental group samples and were excluded from the analysis.

### RNA isolation and cDNA synthesis

Liver RNA isolation was performed by using the RNeasy Microarray Tissue Mini Kit (Qiagen, USA) following the manufacturer’s protocol. Briefly, 30 mg of tissue samples were homogenized using tissue ruptor and lysis buffer. The lysate was precipitated with 70% ethanol and washed twice with washing buffer. Finally the RNA pellet was dissolved in 50 μl of RNase-free water. The isolated RNA was run through NanoDrop spectrophotometer (Thermo Fisher Scientific, USA) to determine both RNA yield and purity by measuring absorbance at 260 and 280 nm. The RNA was reversely transcribed to complementary DNA sequence (cDNA) by using the RT^2^ First Strand Kit (Qiagen, USA) according to manufacturer’s protocol. A starting amount of 1.5 μg of total RNA was used for the reverse transcription process. In brief, the genomic DNA elimination reactions were performed in a final volume of 10 μl using the buffer provided by the manufacturer and incubated at 42 °C for 5 min. Then, 10 μl of the reverse-transcription mix were added into each tube, followed by incubation at 42 °C for 15 min and 95 °C for 5 min. The amplified cDNA was then diluted with nuclease-free water.

### Real-time PCR and gene expression analysis

The level of gene expression in the liver was analyzed by using the Mouse Inflammatory Cytokines & Receptors RT^2^ Profiler™ PCR Array (PAMM-011Z; Qiagen, USA), which profiles the expression of 84 key genes encoding the inflammatory cytokines and receptors. The PCR mix was performed with the following mixture: 1350 μl of 2X SYBR Green Master Mix, 102 μl first-strand cDNA synthesis reaction and 1248 μl of RNase-free water. A total of 25 μl of the cocktail was added into each well of the RT^2^ Profiler PCR array plate.The real-time qPCR was performed using a 7500 Fast Real-Time PCR System (Applied Biosystems, USA) under the following thermal cycle condition: a start cycle for 10 min at 95 °C, 40 cycles of amplification (15 s at 95 °C, 1 min at 60 °C) followed by a melt curve. The Ct values of the target cDNAs were normalized by the average Ct of 5 housekeeping genes (ACTB, B2M, GAPDH, GUSB and HSP90AB1) within the same plate. The PCR array was performed in singleton reaction for each sample.

### Statistical analysis

For plasma cytokine, an ANOVA comparison analysis was done by using GraphPad Prism 7.0 Software. For gene expression analysis, data analysis was conducted using online RT^2^ Profiler PCR Array data analysis software provided by the manufacturer. The software used the ∆∆ Ct method to calculate the fold change between the experimental groups. Statistical analysis was done using two-tailed Student’s t-test. *P*-value < 0.05 was considered statistically significant difference.

## Results

### HPLC fingerprint analysis of FCPLJ

**Fig. 1:**
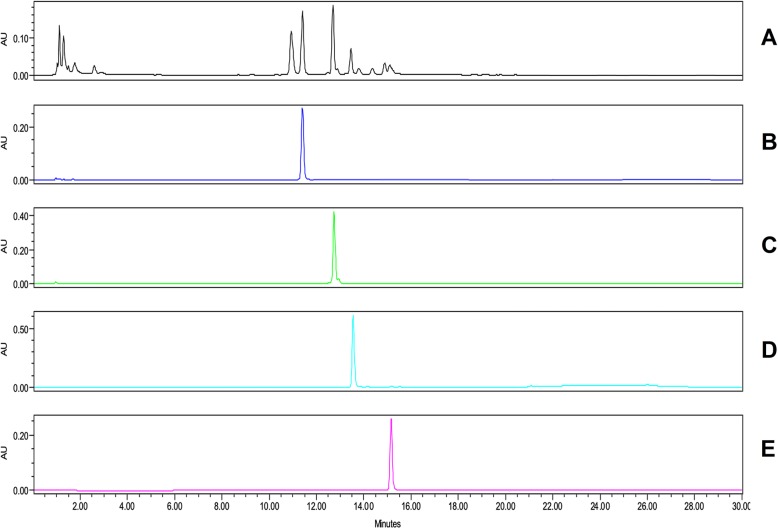
HPLC chromatograms for **a.** freeze-dried *C. papaya* leaf juice (FCPLJ) **b.** manghaslin, **c.** clitorin **d.** rutin and **e.** nicotiflorin

Four peaks of major compounds were determined in the FCLPJ by HPLC (Fig. 1). These compounds were identified as manghaslin, clitorin, rutin and nicotiflorin by using standard with retention time of 11.50, 12.53, 13.50 and 15.10, respectively (Table 1).

**Table 1:** Compound identification in freeze-dried *Carica papaya* leaf juice

Compounds	Molecular formula	Molecular weight, gmol^− 1^	Retention time (RT), min
Manghaslin	C_33_H_40_O_20_	756.66	11.50
Clitorin	C_33_H_40_O_19_	740.66	12.53
Rutin	C_27_H_30_O_16_	610.52	13.50
Nicotiflorin	C_27_H_32_O_15_	596.53	15.10

### Effect of FCPLJ treatment on plasma NS1 level

**Fig. 2:**
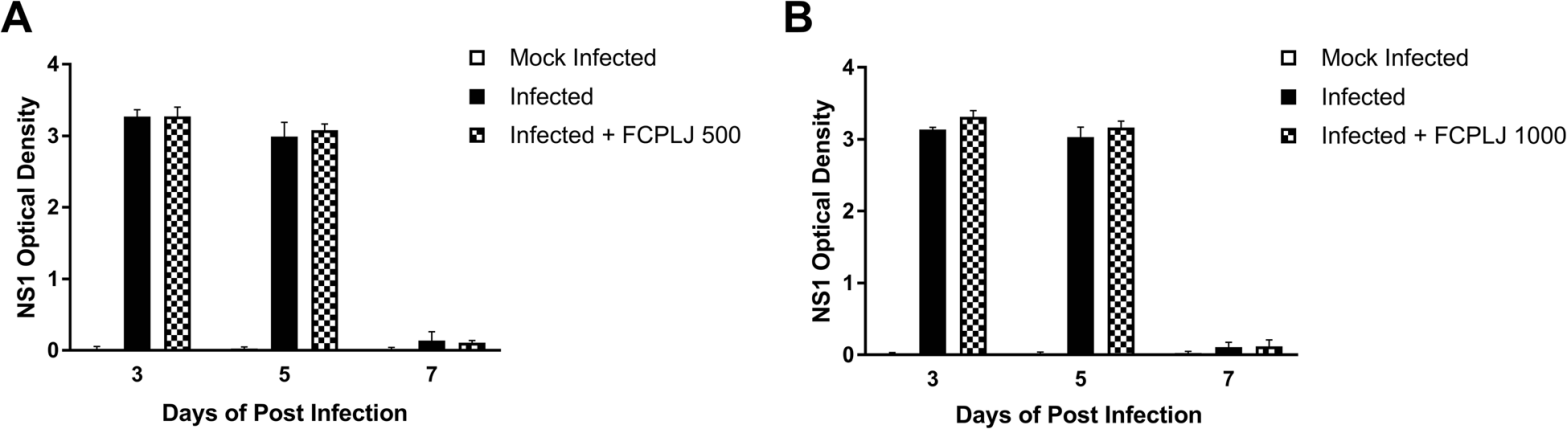
NS1 level of AG129 mice infected with dengue virus strain NGC (2X10^6^ PFU) on day 3, 5 and 7 of post infection detected by NS1 antigen immunoassay. The NS1 levels in plasma of mock infected (white bars), infected (black bars) and infected + FCPLJ (checkered bars) **a**. NS1 level in plasma of AG129 mice treated with 500 mg/kg BW of FCPLJ. **b**. NS1 level in plasma of AG129 mice treated with 1000 mg/kg BW of FCPLJ. There was no significant difference between observed groups

All AG129 mice from infected groups started developing viremia after the inoculation of dengue virus. The dengue virus in the infected group was confirmed by the presence of high NS1 level in the plasma. The plasma NS1 level of the infected AG129 mice started to peak on day 3 post infection and began to decline in between day 5 and day 7 post infection (Fig. 2). The mock infected group showed negligible level of plasma NS1. High level of plasma NS1 was also observed in the FCPLJ treated group, suggesting that the FCPLJ treatment has no effect on the plasma NS1 level in the infected AG129 mice. Similar NS1 level between infected and treatment groups was also observed in the gene expression animal group euthanized on day 4 of post infection (Additional file 1: Figure S1).

### Effect of FCPLJ treatment on leukocyte, cytokine and chemokine responses in AG129 mice infected with dengue virus

**Fig. 3:**
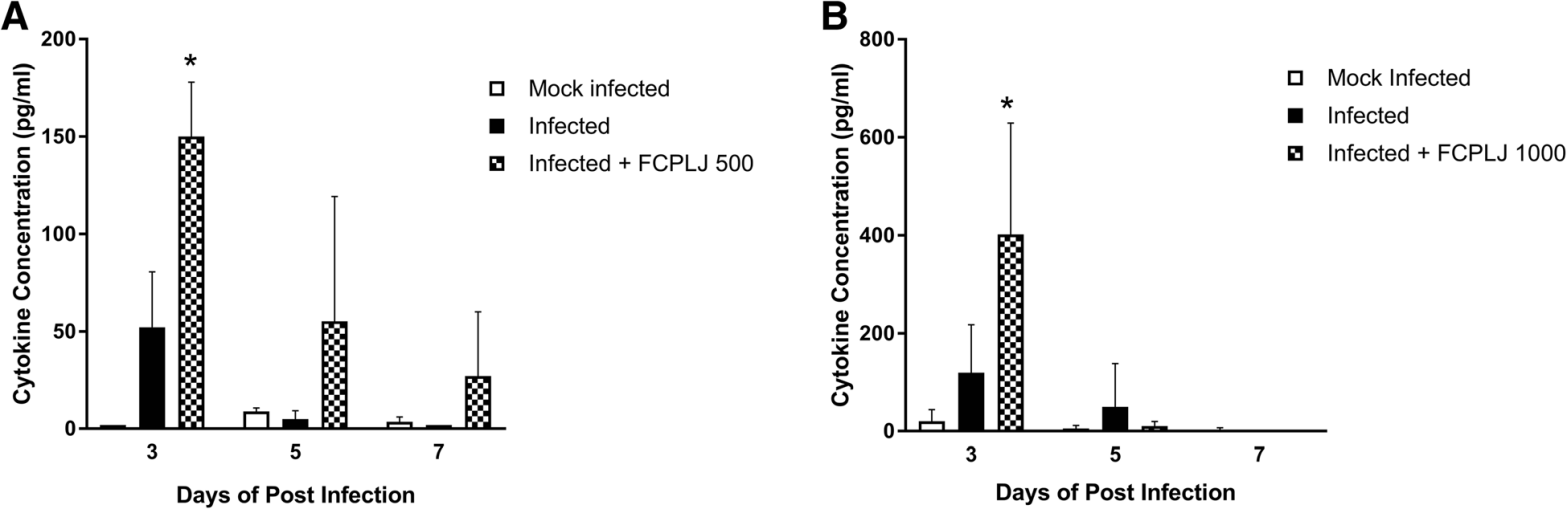
The freeze-dried *C. papaya* leaf juice (FCPLJ) increased the monocyte chemoattractant protein 1 (CCL2/MCP-1) in the plasma of AG129 mice infected with dengue virus. The CCL/MCP-1 cytokine level in plasma collected from mock infected (white bars), infected (black bars) and infected + FCPLJ (checkered bars) AG129 mice groups at day 3, day 5 and day 7 post-infection were analyzed by Procartaplex immunoassay. Bars represent the mean values ± SEM. The cytokine level was compared between experimental groups using ANOVA multiple comparison test. **a.** CCL/MCP-1 level in plasma of AG129 mice treated with 500 mg/kg BW of FCPLJ. **b.** CCL/MCP-1 level in plasma of AG129 mice treated with 1000 mg/kg BW of FCPLJ. Astrisk (*) denotes as significant difference at *P* value < 0.05

The leukocyte count in AG129 mice was increased during the dengue virus infection. In addition, the neutrophil percentage was increased while the lymphocyte percentage was decreased during dengue virus infection (Additional file 2: Table S1). However, the FCPLJ treatment (500 and 1000 mg/kg BW) did not significantly affect the leukocyte’s level as compared to infected group. The effect of FCPLJ treatment on cytokines level was investigated in AG129 mice on day 3, day 5 and day 7 post-infection. The results showed that the level of inflammatory cytokines (G-CSF, IFN-γ, IL-6, IL-18, MCP-1 and TNF-α) in infected group were higher as compared to mock infected AG129 mice group (Fig. 3 and Additional files 3 and 4: Figure S2-S3). The treatment of FCPLJ (500 and 1000 mg/kg BW) has significantly increased MCP-1 level (*p* < 0.05) (Fig. 3). Other cytokines such as G-CSF, IL-6, and TNF-α were apparently increased by FCPLJ treatment especially on day 3 post infection (Additional files 3 and 4: Figure S2 and S3).

### Effect of FCPLJ treatment on gene expression profile of inflammatory cytokines and receptors in the liver of AG129 mice infected with dengue virus

**Fig. 4:**
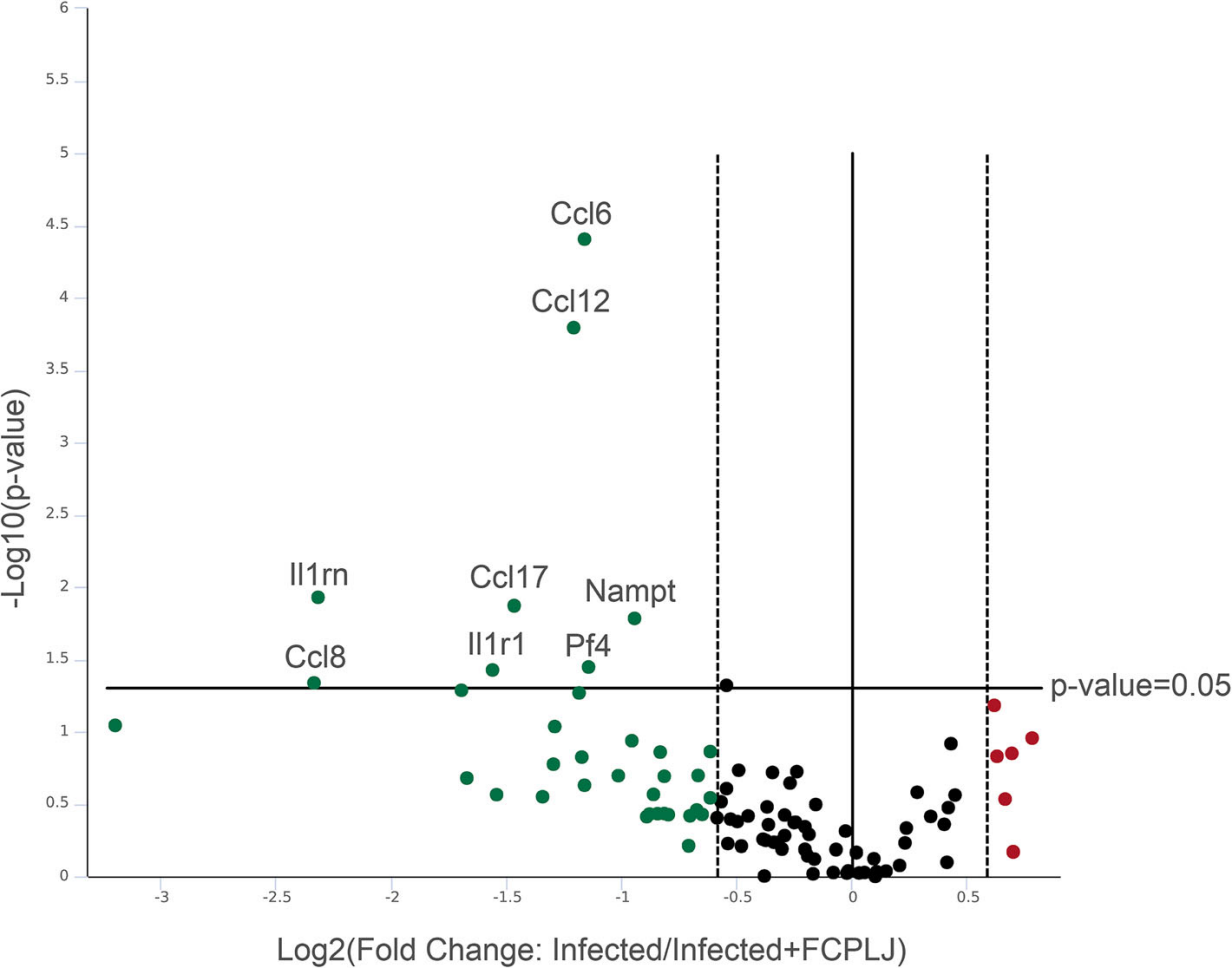
The freeze-dried *C. papaya* leaf juice (FCPLJ) effects on gene expression of inflammatory cytokines and receptors in the liver of AG129 mice infected with dengue virus on day 4 of post-infection. The volcano plot showing the fold change for each of the 84 genes in the array: upregulated (red dots), unchanged (black dots) and downregulated (green dots). Genes, which were significantly downregulated (*P* < 0.05) in infected + FCPLJ group in comparison with infected group, were indicated

As compared to mock infected group, a total of 26 genes were upregulated in the liver of AG129 mice infected with dengue virus (Additional file 5: Table S2). The number of upregulated genes were decreased to 22 genes in the liver of FCPLJ treated AG129 mice infected with dengue virus. As compared to the infected group, there was a significant downregulation of 8 genes in the liver of FCPLJ treated AG129 mice infected with dengue virus. These genes were CCL6/MRP-1, CCL8/MCP-2, CCL12/MCP-5, CCL17/TARC, IL1R1, IL1RN/IL1Ra, NAMPT/PBEF1 and PF4/CXCL4 (Fig. 4 and Table 2).

**Table 2:** Cytokines and receptors showing significant differential gene expression level in the liver of infected AG129 mice treated with FCPLJ^a^ compared to infected AG129 mice without treatment

Gene target	Description	Fold regulation^b^	*P* value^c^
*CCL12*	Chemokine (c-c motif) ligand 12	−2.31	0.000161
*CCL17*	Chemokine (c-c motif) ligand 17	−2.76	0.013469
*CCL6*	Chemokine (c-c motif) ligand 6	−2.23	0.000039
*CCL8*	Chemokine (c-c motif) ligand 8	−5.05	0.045934
*IL1R1*	Interleukin-1 receptor, type 1	−2.95	0.037396
*IL1RN*	Interleukin-1 receptor antagonist	−4.99	0.011812
*NAMPT*	Nicotinamide phosphoribosyltransferase	−1.92	0.016427
*PF4*	Platelet factor 4	−2.21	0.035662

^a^Freeze-dried *Carica papaya* leaf juice; dengue virus infected AG129 mice were given 1000 mg/kg BW

^b^The fold regulation cut off is 1.5

^c^Gene expression are considered to show significant differential expression at a *P* value of < 0.05

## Discussion

Four flavonoid compounds; manghaslin with retention time at 11.50 min, clitorin at 12.53 min, rutin at 13.50 min and nicotiflorin at 15.10 min were identified in FCPLJ samples. The retention time of each compound was decreased along with high molecular weight of the compound corresponding to diverse range of adsorption affinities. Several studies have identified these flavonoid compounds in FCPLJ, in which manghaslin and clitorin were found to be highly abundant flavonoid compounds in the leaves [19–21, 26]. These four compounds were also found to be high in butanol fraction compared to methanol extract of FCPLJ samples [21]. It has been reported that flavonoids have potent immunomodulatory activities and antiviral properties [27–29].

In the present study, AG129 mice in the infected group were inoculated with 2X10^6^ PFU dengue virus DEN-2. The infection was confirmed by assessing the NS1 level in plasma samples. Our initial observation found that the NS1 level started to peak at day 3 of post infection and was almost absent at day 7 of post infection. It has been reported that serum or plasma dengue virus NS1 level correlates with viremia titer and severity of the disease [30, 31]. Treatment with FCPLJ in this study does not stop the progress of viremia development, therefore suggesting that FCPLJ does not have direct effect on the virus.

The possible in vivo immunomodulatory effect of FCPLJ was detected in the present study by the increase in plasma cytokines particularly the CCL2/ MCP-1 in dengue virus infected AG129 mice treated with 500 mg/kg BW and 1000 mg/kg BW FCPLJ. The importance of CCL2/MCP-1 in dengue infection has been reported in many studies involving AG129 dengue mouse model where the cytokine was found to be increased during infection [24, 32–34]. The CCL2/MCP-1 has its role in macrophage and monocyte recruitments to the site of infection. This molecule is produced by a variety of cell types including fibroblasts, cardiomyocytes, endothelial and smooth muscle cells, and monocytic cells; either constitutively or after induction by oxidative stress, cytokines and growth factors [35]. In dengue infection, platelet-monocyte aggregation was induced in dengue patients and the aggregate formation could enhanced the production of cytokines and chemokines by monocytes including TNF-α, IL-1b, IL-8, and CCL2/MCP-1 [36].

Histopathological changes in liver of human dengue fatal cases have been reported [37–39]. Many in vitro studies have demonstrated that the infection of dengue virus in hepatoma cell lines [40–42] lead to important dengue-host interaction; including stimulation of innate immunity, apoptosis, induction of cytokine and chemokine [43]. We assessed the effect of FCPLJ on the induction of inflammatory cytokine gene expression in liver during dengue virus infection in AG129 mice. Our study revealed a significant upregulation of 26 genes in the liver following the infection with DEN2 in AG129 mice compared to healthy control group. Treatment with 1000 mg/kg BW FCPLJ in infected group showed a decrease number of significant upregulated genes to 22. Similar with the elevated level of CCL2/MCP-1 level in plasma cytokine on day 3, upregulation of CCL2/MCP-1 was also observed in the liver of treated group (Infected+FCPLJ) as compared to infected group, although the increase was not significant (Table S2).

As compared to the infected group, the FCPLJ treatment showed significant downregulation of these 8 genes; CCL6/MRP-1, CCL8/MCP-2, CCL12/MCP-5, CCL17/TARC, IL1R1, IL1RN/IL1Ra, NAMPT/PBEF1 and PF4/CXCL4. CCL6/MRP-1, CCL12/MCP-5, and IL1R1 have not been previously described in dengue virus infections in vitro or in patients. A strong stimulation of CCL8/MCP-2 has been reported in human primary monocytes, B cells and dendritic cells infected with dengue virus in vitro [44]. Due to its various binding receptors such as CCR1, CCR2 and CCR5, CCL8/MCP-2 may have independent effects on immune response to dengue virus infection by engaging with different receptors to activate or suppress the effect of other chemokines. The upregulation of CCL17/TARC was reported in the spleen of dengue virus infected mice [45]. Elevated levels of anti-inflammatory cytokine IL1RN/IL1Ra was previously observed in dengue patients [46, 47] and IL1RN/IL1Ra level was higher in dengue with the warning signs patients compared to the dengue without the warning signs patients [48]. Downregulation of PF4/CXCL4 gene by two folds following the treatment with FCPLJ in dengue infected mice in this study showed that FCPLJ could has suppressed the expression of PF4/CXCL4 gene to increase platelet production during dengue infection. There was an evidence showing that PF4/CXCL4 as an in vivo negative autocrine regulator of platelet production [49]. Furthermore, it was reported that PF4/CXCL4 was increased in plasma of dengue patients compared to healthy volunteers [50]. The upregulation of NAMPT/PBEF1 in dengue virus infected mice was in concurrence with the finding of previous in vivo study [51]. The function of NAMPT/PBEF1 as an inflammatory cytokine has been implicated in other acute and chronic inflammatory conditions [52] and has been shown to upregulate several prominent inflammatory cytokines such as IL-6, TNF-α, IL-1β, and, more recently, TGF-β1 [53].

Previous studies have reported a relationship between high levels of pro-inflammatory cytokine production with severity of the dengue disease. [54, 55]. Although the mechanism of the disease pathogenesis has not been clarified, it has been implied that the released of cytokines and other mediators during the immune response to dengue virus infection, may contribute for the increased permeability of vascular endothelial cells [44, 56]. Therefore, the effect of FCPLJ treatment to downregulate certain genes as demonstrated in this study suggested that FCPLJ may serve as potential immunomodulator of host immune responses during dengue virus infection.

In this study, although all samples were accounted and tested for cytokine levels, certain cytokines were undetectable in some samples. The study focused on the plasma and liver cytokines. Other organs such as spleen, kidney, heart, lung and brain were not included in our analysis. Therefore, we might leave out what could be important informations of FCPLJ effect on cytokines level in other vital organs. This study could not highlight the functional activities of the affected cytokines. In order to validate the finding of this study, further analysis such as histology stain of the liver, protein expression or immunohistochemistry analysis need to be conducted in future studies. As the FCPLJ has been proven as a thrombocytosis agent, the information on its mechanism of action in enhancing platelet production and immunomodulatory activity during dengue infection could be interesting subject to explore further.

## Conclusion

The present study has generated an initial data highlighting the possible function of FCPLJ in modulating the release of certain cytokines during dengue infection in infected AG129 mice specifically the CCL2/MCP-1 level during peak of viremia. FCPLJ treatment has also downregulated inflammatory cytokine genes such as CCL6/MRP-1, CCL8/MCP-2, CCL12/MCP-5, CCL17/TARC, IL1R1, IL1RN/IL1Ra, NAMPT/PBEF1 and PF4/CXCL4 in the liver of infected AG129 mice. More studies are still necessary to address the specific immunomodulatory role played by FCPLJ during dengue infection.

## Additional files

Additional file 1: **Figure S1.** NS1 level of AG129 mice infected with dengue virus (2X10^6^ PFU) on day 4 of post infection detected by NS1 antigen immunoassay. (PDF 58 kb)

Additional file 2: **Table S1.** Total white blood cells and differential counts of AG129 mice infected with dengue virus. (PDF 39 kb)

Additional file 3: **Figure S2.** The cytokine level in plasma of dengue virus infected AG129 mice treated with 500 mg/kg BW of FCPLJ. (PDF 151 kb)

Additional file 4: **Figure S3.** The cytokine level in plasma of dengue virus infected AG129 mice treated with 1000 mg/kg BW of FCPLJ. (PDF 133 kb)

Additional file 5: **Table S2**. Fold Regulation of 84 genes associated with mouse inflammatory cytokines & receptors. (PDF 75 kb)

## Abbreviations

IFN-γ: Interferon gamma
DMEM: Dulbecco’s modified eagle’s medium
ACTB: Actin B
ANOVA: Analysis of variance
B2M: Beta-2-microglobulin
BW: Body weight
CCL12/MCP-5: Chemokine (c-c motif) ligand 12/monocyte chemoattractant protein-5
CCL17/TARC: Chemokine (c-c motif) ligand 17/thymus and activation-regulated chemokine
CCL2/MCP-1: Chemokine (c-c motif) ligand 2/monocyte chemoattractant protein-1
CCL3/MIP-1A: Chemokine (c-c motif) ligand 3/macrophage inflammatory protein 1-alpha
CCL6/MRP-1: Chemokine (c-c motif) ligand 6/multidrug resistance-associated protein 1
CCL8/MCP-2: Chemokine (c-c motif) ligand 8/monocyte chemoattractant protein-2
cDNA: Complementary DNA sequence
CPLJ: *C. papaya* leaf juice
CSF2/GM-CSF: Colony stimulating factor 2/granulocyte-macrophage colony-stimulating factor
CSF3/G-CSF: Colony stimulating factor 3/granulocyte-colony stimulating factor
DEN-2: Dengue virus type
EDTA: Ethylenediaminetetraacetic acid
FCPLJ: Freeze-dried *C. papaya* leaf juice
GAPDH: Glyceraldehyde-3-phosphate dehydrogenase
GUSB: Glucuronidase beta
HPLC: High performance liquid chromatography
HSP90AB1: Heat shock protein 90 alpha family class b member
I IL1RN/IL1Ra: Interleukin-1 receptor antagonist
IL-10: Interleukin 10
IL-13: Interleukin 13
IL-18: Interleukin 18
IL1R1: Interleukin-1 receptor type
IL-1β: Interleukin 1, beta
IL-4: Interleukin 4
IL-6: Interleukin-6
NAMPT/PBEF1: Nicotinamide phosphoribosyltransferase/pre-b-cell colony-enhancing factor 1
NS1: Nonstructural protein 1
PBMCs: Peripheral blood mononuclear cells
PCR: Polymerase chain reaction
PF4/CXCL4: Platelet factor 4/chemokine (c-x-c motif) ligand 4
PFU: Plaque forming units
RNA: Ribonucleic acid
TFA: Trifluoroacetic acid
TNF-α: Tumor necrosis factor alpha
