# Supplementary material for: Effect of freeze-dried Carica papaya leaf juice on inflammatory cytokines production during dengue virus infection in AG129 mice
Source: BMC Complement Altern Med. 2019 Feb 11;19:44. doi: 10.1186/s12906-019-2438-3 (PMC6371484; doi:10.1186/s12906-019-2438-3)
Supplement: Supplementary file 1 — Figure S1. NS1 level of AG129 mice infected with dengue virus (2X106 PFU) on day 4 of post infection detected by NS1 antigen immunoassay. (PDF 58 kb) [file 12906_2019_2438_MOESM1_ESM.pdf]

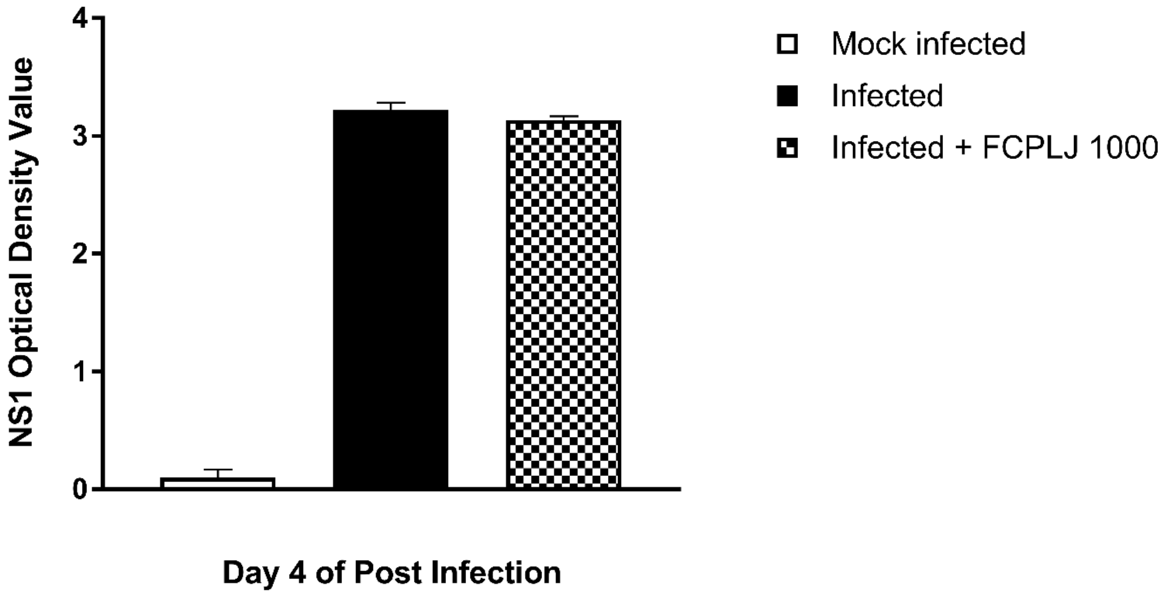

Figure S1. NS1 level of AG129 mice infected with dengue virus (2X10^{6} PFU) on day 4 of post infection detected by NS1 antigen immunoassay. The NS1 levels in plasma of mock infection (white bars), infected (black bars) and infected + FCPLJ (checkered bars) AG129 mice groups. The treated group was given 1000 mg/kg BW of FCPLJ. There was no significant difference between observed groups.
