# Supplementary material for: Effect of freeze-dried Carica papaya leaf juice on inflammatory cytokines production during dengue virus infection in AG129 mice
Source: BMC Complement Altern Med. 2019 Feb 11;19:44. doi: 10.1186/s12906-019-2438-3 (PMC6371484; doi:10.1186/s12906-019-2438-3)
Supplement: Supplementary file 2 — Table S1. Total white blood cells and differential counts of AG129 mice infected with dengue virus. (PDF 39 kb) [file 12906_2019_2438_MOESM2_ESM.pdf]

Table S1. Total white blood cells and differential counts of AG129 mice infected with dengue virus.

Experimental Group	Mock Infection			Infected Control			Infected + FCPLJ (500 mg/kg BW)		
Day of Post Infection	3	5	7	3	5	7	3	5	7
Total White Blood Cell Count ($X10^3$/uL)	2.28±0.22	2.44±0.07	3.36±0.50	3.24±0.12	4.80±0.54	4.56±1.07	3.32±0.39	4.44±0.36	3.68±0.43
Neutrophil (%)	9.40±1.52	6.40±2.30	7.40±1.14	45.20±9.26	36.00±8.19	25.40±5.32	37.60±5.03	38.20±5.07	25.60±5.81
Lymphocyte (%)	87.40±4.51	91.20±3.03	90.20±1.30	51.40±7.16	60.40±8.96	73.20±4.92	57.80±5.59	60.00±5.92	73.40±6.54

Experimental Group	Mock Infection			Infected Control			Infected + FCPLJ (1000 mg/kg BW)		
Day of Post Infection	3	5	7	3	5	7	3	5	7
Total White Blood Cell Count ($X10^3$/uL)	3.12±0.15	3.92±0.71	2.80±0.14	5.12±0.39	8.68±0.78	9.48±0.64	4.00±0.30	8.32±1.49	8.28±0.50
Neutrophil (%)	13.20±4.23	11.20±3.90	12.60±6.73	39.60±3.78	37.20±7.66	26.20±3.42	39.20±3.70	35.20±7.89	29.60±3.91
Lymphocyte (%)	84.60±5.41	87.20±4.44	85.20±6.46	56.40±3.85	55.40±6.99	67.40±3.29	56.20±3.90	58.20±5.54	65.80±3.42
