# Supplementary material for: Effect of freeze-dried Carica papaya leaf juice on inflammatory cytokines production during dengue virus infection in AG129 mice
Source: BMC Complement Altern Med. 2019 Feb 11;19:44. doi: 10.1186/s12906-019-2438-3 (PMC6371484; doi:10.1186/s12906-019-2438-3)
Supplement: Supplementary file 4 — Figure S3. The cytokine level in plasma of dengue virus infected AG129 mice treated with 1000 mg/kg BW of FCPLJ. (PDF 133 kb) [file 12906_2019_2438_MOESM4_ESM.pdf]

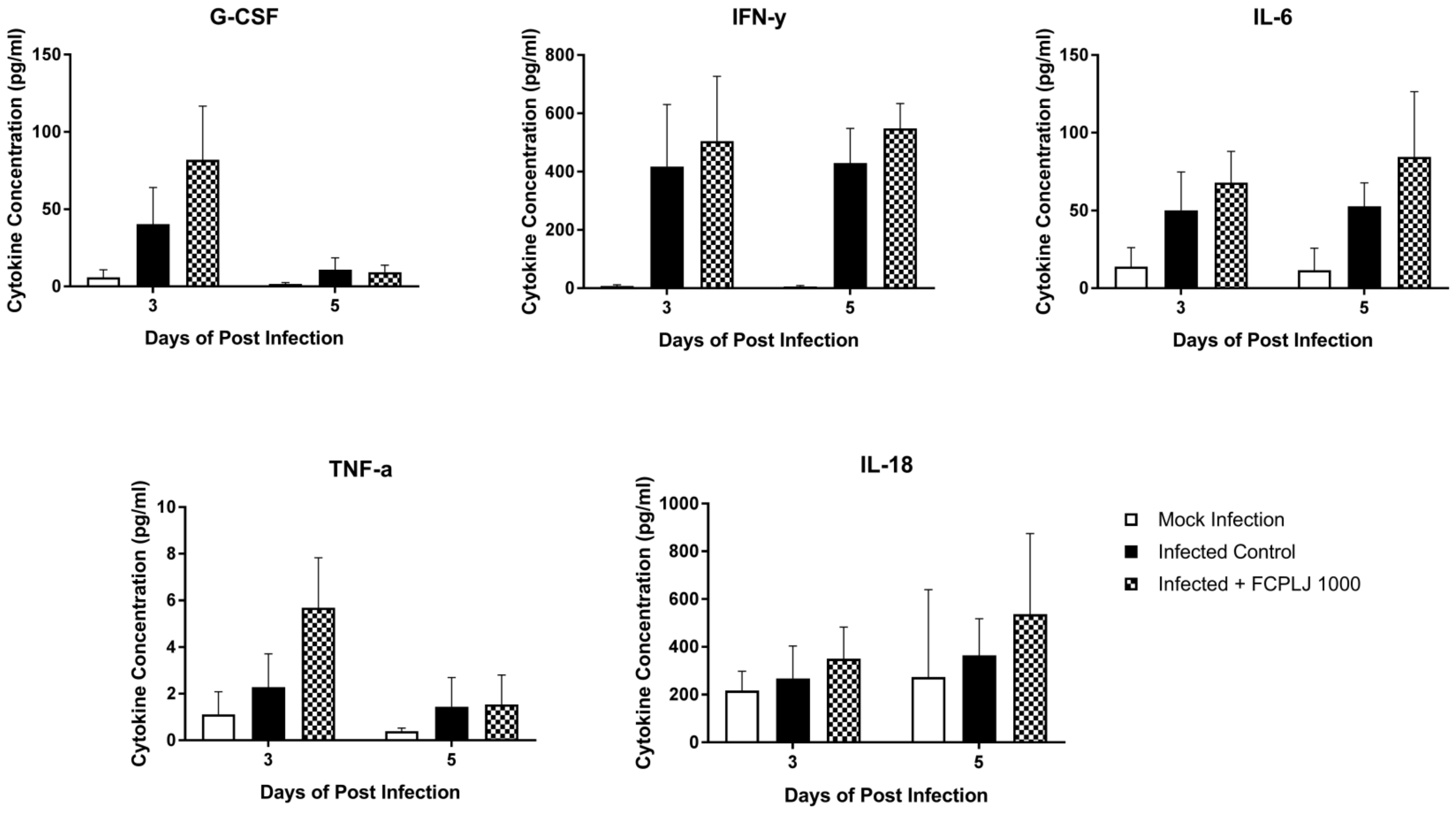

Figure S3. The cytokine level in plasma collected from mock infection (white bars), infected (black bars) and infected + FCPLJ (checkered bars) AG129 mice groups at day 3 and day 5 post-infection were analyzed by Procartaplex immunoassay. The treated group was given 1000 mg/kg BW of FCPLJ. Bars represent the mean values ± SEM. The cytokine level was compared between experimental groups using ANOVA multiple comparison test. There was no significant difference between observed groups.
