# Supplementary material for: Effect of freeze-dried Carica papaya leaf juice on inflammatory cytokines production during dengue virus infection in AG129 mice
Source: BMC Complement Altern Med. 2019 Feb 11;19:44. doi: 10.1186/s12906-019-2438-3 (PMC6371484; doi:10.1186/s12906-019-2438-3)
Supplement: Supplementary file 5 — Table S2. Fold Regulation of 84 genes associated with mouse inflammatory cytokines & receptors. (PDF 75 kb) [file 12906_2019_2438_MOESM5_ESM.pdf]

Table S2. Fold Regulation of 84 genes associated with mouse inflammatory cytokines & receptors.

Gene Symbol	Gene Name	Up-Down Regulation (comparing to mock infected mice)			
		Infected Control		Infected + FCPLJ	
		Fold Regulation	p-value	Fold Regulation	p-value
Aimp1	Aminoacyl tRNA synthetase complex-interacting multifunctional protein 1	1.6541	0.042196	1.3046	0.24001
Bmp2	Bone morphogenetic protein 2	-1.5783	0.223831	1.0888	0.909343
Ccl1	Chemokine (C-C motif) ligand 1	1.088	0.865433	1.1037	0.832525
Ccl11	Chemokine (C-C motif) ligand 11	4.0341	0.149335	2.1719	0.214682
Ccl12	Chemokine (C-C motif) ligand 12	22.6242	0.000007	9.7765	0.001621
Ccl17	Chemokine (C-C motif) ligand 17	11.9717	0.001217	4.3306	0.026254
Ccl19	Chemokine (C-C motif) ligand 19	7.7644	0.011255	3.4199	0.002748
Ccl2	Chemokine (C-C motif) ligand 2	18.5032	0.000831	21.7947	0.001205
Ccl20	Chemokine (C-C motif) ligand 20	1.6057	0.416977	-1.014	0.722438
Ccl22	Chemokine (C-C motif) ligand 22	1.4884	0.353473	-1.0299	0.84382
Ccl24	Chemokine (C-C motif) ligand 24	2.4705	0.069496	1.005	0.574089
Ccl3	Chemokine (C-C motif) ligand 3	5.0431	0.014775	2.0644	0.259604
Ccl4	Chemokine (C-C motif) ligand 4	2.0044	0.194833	1.3498	0.148236
Ccl5	Chemokine (C-C motif) ligand 5	2.0814	0.007265	1.7257	0.0016
Ccl6	Chemokine (C-C motif) ligand 6	17.1368	0.000001	7.6807	0.000017
Ccl7	Chemokine (C-C motif) ligand 7	14.218	0.000602	11.6333	0.005276
Ccl8	Chemokine (C-C motif) ligand 8	8.9448	0.034124	1.7698	0.400712
Ccl9	Chemokine (C-C motif) ligand 9	1.2467	0.495616	-1.2297	0.492467
Ccr1	Chemokine (C-C motif) receptor 1	14.5391	0.0032	18.4307	0.00011
Ccr10	Chemokine (C-C motif) receptor 10	-1.3079	0.682978	1.016	0.808848
Ccr2	Chemokine (C-C motif) receptor 2	4.9775	0.079601	3.8282	0.271377
Ccr3	Chemokine (C-C motif) receptor 3	3.0449	0.059125	2.349	0.129661
Ccr4	Chemokine (C-C motif)	2.867	0.2901	-1.014	0.722438

	receptor 4				
Ccr5	Chemokine (C-C motif) receptor 5	4.3161	0.074304	2.9724	0.258167
Ccr6	Chemokine (C-C motif) receptor 6	2.5854	0.158333	1.4704	0.50784
Ccr8	Chemokine (C-C motif) receptor 8	1.7102	0.402248	-1.014	0.722438
Cd40lg	CD40 ligand	3.0867	0.182088	1.6967	0.445332
Csf1	Colony stimulating factor 1 (macrophage)	1.8771	0.025963	2.2854	0.008538
Csf2	Colony stimulating factor 2 (granulocyte-macrophage)	1.4472	0.457794	2.3516	0.214685
Csf3	Colony stimulating factor 3 (granulocyte)	2.453	0.311993	-1.0359	0.680086
Cx3cl1	Chemokine (C-X3-C motif) ligand 1	-1.363	0.409984	-1.1806	0.493059
Cxcl1	Chemokine (C-X-C motif) ligand 1	148.4972	0.062947	16.1988	0.158232
Cxcl10	Chemokine (C-X-C motif) ligand 10	2.3822	0.011774	2.349	0.013235
Cxcl11	Chemokine (C-X-C motif) ligand 11	-1.2115	0.796498	-2.1324	0.160784
Cxcl12	Chemokine (C-X-C motif) ligand 12	1.1405	0.384876	1.1298	0.375924
Cxcl13	Chemokine (C-X-C motif) ligand 13	29.3875	0.015781	9.0682	0.004148
Cxcl15	Chemokine (C-X-C motif) ligand 15	-1.0057	0.581736	-1.6069	0.259065
Cxcl5	Chemokine (C-X-C motif) ligand 5	1.2715	0.872635	1.2019	0.916448
Cxcl9	Chemokine (C-X-C motif) ligand 9	2.3971	0.727837	2.447	0.740416
Cxcr2	Chemokine (C-X-C motif) receptor 2	13.4788	0.004786	11.6943	0.000046
Cxcr3	Chemokine (C-X-C motif) receptor 3	4.4194	0.000205	3.9285	0.030267
Cxcr5	Chemokine (C-X-C motif) receptor 5	1.2296	0.722098	-1.5779	0.172576
Fasl	Fas ligand (TNF superfamily, member 6)	1.9914	0.318988	1.2673	0.791222
Ifng	Interferon gamma	13.7625	0.0508	6.829	0.007452
Il10ra	Interleukin 10 receptor, alpha	1.8333	0.052816	1.9006	0.03264
Il10rb	Interleukin 10 receptor, beta	1.442	0.195524	1.25	0.442146
Il11	Interleukin 11	1.2694	0.565156	-1.014	0.722438
Il13	Interleukin 13	1.9196	0.3545	1.6138	0.564409
Il15	Interleukin 15	-1.4926	0.213081	-1.1176	0.737156
Il16	Interleukin 16	1.1325	0.944547	1.219	0.884378
Il17a	Interleukin 17A	-1.0894	0.734313	-1.014	0.722438
Il17b	Interleukin 17B	1.4779	0.448147	-1.014	0.722438
Il17f	Interleukin 17F	-1.3089	0.697741	1.2106	0.296719
Il1a	Interleukin 1 alpha	1.3521	0.524064	1.8411	0.136373
Il1b	Interleukin 1 beta	1.4034	0.096977	2.1504	0.005153

Il1r1	Interleukin 1 receptor, type I	13.1076	0.01163	4.4435	0.000248
Il1rn	Interleukin 1 receptor antagonist	47.8763	0.004371	9.6001	0.005489
Il21	Interleukin 21	1.12	0.856458	-1.014	0.722438
Il27	Interleukin 27	2.018	0.308132	-1.1088	0.595659
Il2rb	Interleukin 2 receptor, beta chain	2.9675	0.041689	3.4869	0.145283
Il2rg	Interleukin 2 receptor, gamma chain	3.9965	0.00005	3.27	0.006171
Il3	Interleukin 3	1.631	0.402537	-1.1015	0.622102
Il33	Interleukin 33	2.993	0.058094	1.6811	0.048169
Il4	Interleukin 4	1.8132	0.331056	-1.7583	0.114099
Il5	Interleukin 5	2.0272	0.243264	1.9901	0.273847
Il5ra	Interleukin 5 receptor, alpha	-1.134	0.705699	-1.0222	0.740907
Il6ra	Interleukin 6 receptor, alpha	1.7504	0.200258	1.3534	0.435067
Il6st	Interleukin 6 signal transducer	2.4899	0.020905	1.7679	0.088152
Il7	Interleukin 7	3.767	0.012557	2.5811	0.029
Lta	Lymphotoxin A	1.8168	0.402671	-1.014	0.722438
Ltb	Lymphotoxin B	2.2383	0.194709	2.1308	0.067371
Mif	Macrophage migration inhibitory factor	1.0327	0.869322	-1.4124	0.28265
Nampt	Nicotinamide phosphoribosyltransferase	2.8771	0.002438	1.4962	0.088577
Osm	Oncostatin M	2.0764	0.269921	1.8553	0.331594
Pf4	Platelet factor 4	2.8685	0.010246	1.3003	0.283835
Spp1	Secreted phosphoprotein 1	2.1738	0.248424	-1.0378	0.668161
Tnf	Tumor necrosis factor	1.3462	0.434461	1.1365	0.908904
Tnfrsf11b	Tumor necrosis factor receptor superfamily, member 11b	1.9013	0.031263	2.0301	0.023201
Tnfsf10	Tumor necrosis factor (ligand) superfamily, member 10	-1.5091	0.219302	-1.1441	0.502449
Tnfsf11	Tumor necrosis factor (ligand) superfamily, member 11	1.6653	0.461005	1.2165	0.979051
Tnfsf13	Tumor necrosis factor (ligand) superfamily, member 13	2.9277	0.013706	2.0735	0.168057
Tnfsf13b	Tumor necrosis factor (ligand) superfamily, member 13b	2.029	0.082243	1.774	0.180965
Tnfsf4	Tumor necrosis factor (ligand) superfamily, member 4	1.4198	0.469465	-1.014	0.722438
Vegfa	Vascular endothelial growth factor A	-1.0484	0.936305	1.5442	0.065259
